# Interspecific Larval Competition of Two *Diabrotica* Species (Northern and Western Corn Rootworm) in Corn Roots: Implications for Pest Management

**DOI:** 10.3390/plants15030367

**Published:** 2026-01-24

**Authors:** David S. Wangila, Yucheng Wang, Adrian J. Pekarcik, Fei Yang

**Affiliations:** 1 Department of Entomology, University of Minnesota, Saint Paul, MN 55108, USA; 2 North Central Agricultural Research Laboratory, USDA-Agricultural Research Service, Brookings, SD 57006, USA

**Keywords:** maize, corn rootworm, competition, integrated pest management

## Abstract

The western corn rootworm (WCR) and northern corn rootworm (NCR) are the two major belowground insect pests of corn in the U.S. Corn Belt. These species coexist in the same habitat, where their larvae feed on corn roots, increasing the risk of lodging and yield loss. Understanding larval competition between WCR and NCR is crucial for effective insect resistance management and integrated pest management. To assess interspecific larval competition between WCR and NCR, two independent greenhouse trials were conducted. We infested non-Bt corn plants with varying egg ratios of diapause and non-diapause populations of both species and counted the number of adults of each species recovered from each plant. Results showed that WCR consistently exhibited higher emergence rates than NCR, regardless of the initial egg infestation ratio. The observed ratio of NCR to WCR in both diapause and non-diapause groups was significantly lower than expected, suggesting that WCR is more competitive than NCR. The competitive dominance of WCR, coupled with climate warming, may facilitate its northward expansion across the U.S. This could potentially affect local NCR populations and further spread Bt and rotation resistance. Such changes could exacerbate pest management challenges in corn production systems. Integrating knowledge of corn rootworm competition, biology, resistance development, and climate change will be critical for developing informed management strategies to mitigate corn rootworm damage in agroecosystems effectively.

## 1. Introduction

The western corn rootworm (WCR), *Diabrotica virgifera virgifera* LeConte, and the northern corn rootworm (NCR), *Diabrotica barberi* Smith & Lawrence (Coleoptera: Chrysomelidae) are the two major belowground insect pests of corn. Annually, the two pests cost U.S farmers an estimated $2.0 billion in yield losses, economic damage, and management costs [1,2]. While adult WCR primarily feed on corn silks and pollen, NCR adults are less selective and frequently forage on weedy grasses and various broadleaf plants in addition to corn silks and pollen [3]. Larvae of both species feed almost exclusively on corn roots [4]. The larval feeding on corn roots directly impairs the plant’s nutrient and water uptake from the soil. This root injury also increases the risk of lodging and susceptibility to plant pathogens, ultimately leading to yield loss [5,6].

The WCR and NCR have similar biology, with both species being univoltine and overwintering in the soil as eggs [7,8,9]. Egg development starts in spring when soil temperatures exceed the thresholds of 10.5 °C for WCR [10,11] and 10.9 °C for NCR [12]. In addition, post-diapause eggs also need to take up water/soil moisture to complete development [13,14]. Upon hatching, the larvae feed on and tunnel through corn roots [7,15], and males complete larval and pupal development faster than females [16]. Adult emergence typically occurs from mid-summer to early fall [17,18,19], with males emerging earlier than females [20,21]. Females move into the soil through soil cracks or along the brace roots and lay eggs at a depth of about 5–15 cm close to the roots of host plants [22]. Studies have shown that each WCR female can lay about 356–735 eggs, whereas each NCR female can lay only 132–312 eggs, depending on the time of emergence [23]. As environmental temperatures drop below 11 °C, corn rootworm (CRW) eggs enter diapause until post diapause hatching occurs [24].

The WCR and NCR frequently coexist in the same habitat within a landscape [25,26], creating opportunities for inter- and intra-specific larval competition in corn fields. Although larval competition is known to influence CRW behavior, survival, and reproduction, most studies have focused on intraspecific effects, and direct experimental assessments of WCR–NCR interspecific competition remain limited [7,9,27,28,29,30]. The level of larval competition can vary depending on several factors, including the abundance and distribution of food resources, the timing of egg hatch, the density of each species, environmental conditions (temperature, humidity, soil type), and management practices like continuous or rotated corn [9,29,31]. For example, studies have indicated that higher WCR larval densities on corn roots negatively impact emergence and adult size and can also delay development from neonate to adult stage [32,33]. The WCR is widely distributed throughout corn production areas, while NCR is primarily found in the northern regions of the Corn Belt such as Minnesota, Wisconsin, South Dakota, and North Dakota [7,21]. The composition map of NCR and WCR in cornfields reveals distinct dominance of one species in large areas. In regions of Nebraska where continuous corn planting is common, WCR has displaced NCR, while areas with prevalent crop rotation practices show a higher frequency of NCR [34,35]. This pattern has also been observed in South Dakota, where the abundance of WCR but not NCR is positively correlated with proximity of continuous cornfields. NCR exhibits greater local dispersal from cornfields to grass areas with flowering plants and soybean fields, whereas WCR moves between and/or within cornfields [21]. These behavioral differences, coupled with continuous corn planting, partially explain the displacement between WCR and NCR [35]. Together, these landscape-level patterns suggest that interspecific interactions, including larval competition on corn roots, may contribute to shifts in species dominance. This observation provides a rationale for evaluating WCR-NCR larval competition under controlled greenhouse conditions in this study.

Despite substantial progress in understanding CRW life history traits and population dynamics, critical questions remain regarding species-specific adaptations and ecological interactions between these sympatric pests. This knowledge gap is particularly consequential given the recent evolution of CRW resistance to Bt corn in many fields [36]. Although Bt corn hybrids remain effective against many field populations of WCR and NCR, efficacy is highly variable across the Corn Belt [37,38,39,40,41] and these two species exhibit different levels of resistance to Bt traits. Field-evolved resistance to Cry3Bb1 and Gpp34Ab1/Tpp35Ab1 proteins in WCR populations have been frequently documented across the Corn Belt; however, only two published studies have reported field-evolved resistance in NCR to these proteins [36,42,43]. Of particular concern is the consequences of interspecific interactions among larvae in the context of Bt resistance development, coupled with their co-occurrence in certain corn production areas. This includes how these larvae compete, how these interactions may influence larval behavior and how these interactions may affect Bt resistance management. To address these gaps, we conducted two independent greenhouse studies to investigate interspecific competition between NCR and WCR larvae. We hypothesized that the larvae of WCR and NCR differ in their survival under mixed-species conditions. Therefore, we assessed larval competition between WCR and NCR by infesting non-Bt corn plants with varying egg ratios of diapause and non-diapause eggs from both species. The data generated will contribute to the development of more targeted and sustainable pest management strategies for these two economically important pests of corn in the United States.

## 2. Results

### 2.1. Egg Hatch Rate

The overall ANOVA tests showed that egg hatch rates in the laboratory did not differ significantly among the four CRW populations in either trial (Table 1). These results suggest that the observed differences in adult emergence between WCR and NCR populations for either diapause or non-diapause populations in the greenhouse were not due to significant variations in egg hatch rates.

### 2.2. Percentage of Adult Emergence

In trial 1, significant differences in rootworm adult emergence were observed among treatments (*F* = 21.04; df = 9, 82; *p* < 0.0001). The treatment I and J had the highest survival rates. In contrast, the lowest survival rates were observed in treatment A and F (Table 2). The emergence rate for the non-diapause population ranged from 5.13–21.90%, while the diapause population ranged from 6.00–18.25%. Overall, the mean percentage of adult emergence was higher in treatments with greater proportions of WCR eggs, specifically those ranging from 50% to 75% for the diapause population and from 25% to 75% in the non-diapause population (Table 2).

Similarly, significant differences in adult CRW emergence were observed among treatments in trial 2 (*F* = 3.61; df = 9, 70; *p* < 0.001). Emergence rates ranged from 6.75–19.50%, with the lowest emergence recorded in treatment H (6.75%) and the highest in treatment C (19.50%). The percent emergence for the diapause population ranged between 10.80–19.50%, while the non-diapause population ranged from 6.75–14.20%. Within the diapause population, treatments with 50% WCR eggs had the highest emergence rates (Table 2). Among the non-diapause population, those infested with 100% non-diapause WCR eggs had significantly higher emergence rates than the other treatments (Table 2). Overall, increasing the proportion of WCR eggs from 50% to 75% in diapause population led to a corresponding increase in percent emergence (Table 2). However, increasing the proportion of WCR eggs from 25% to 75% in non-diapause population did not result in significant increase in percent emergence (Table 2).

### 2.3. Emergence Ratio

In trial 1, across treatments for both diapause and non-diapause populations, the observed adult emergence ratios of NCR to WCR were significantly lower (*p* < 0.05) than the expected ratio except treatment B, where the observed emergence ratio did not significantly differ from the expected value (*p* = 0.306) (Table 3). Similarly, in trial 2, the observed adult emergence ratios of NCR to WCR for both diapause and non-diapause populations were significantly lower (*p* < 0.05), compared to the expected ratios, except for the treatment G, where no difference was found between the observed and expected ratios of NCR: WCR (*p* = 0.2832) (Table 3). These results suggest that WCR has a competitive advantage over NCR when both species are present as larvae feeding on corn roots (Table 3).

## 3. Discussion

Our study shows that WCR has a competitive advantage over NCR during larval feeding on corn roots, leading to higher survival and emergence rates than NCR across mixed-species treatments. These results support previous findings that WCR outcompetes NCR [9,27,28,44]. In a three-year field study exploring the competitiveness of WCR and NCR, Benkert found that when WCR constituted more than 25% of the initial egg infestation in a mixed population with NCR, adult emergence was disproportionally skewed toward WCR, comprising >50% of emerging beetles [44]. Although egg hatch rates did not differ significantly among populations in our laboratory assays, differences in hatch timing could potentially influence larval establishment. For example, if one species were established earlier, it could have some impact on the establishment of the other species, a scenario that may also occur in the field where both species deposit eggs in the same field. In addition, egg hatch timing for NCR and WCR in the field may vary due to differences in oviposition depth, as soil temperature at varying depths can influence the accumulation of growing degree days and the resumption of post-diapause development. Furthermore, the distribution of eggs laid by different species around specific plants may be non-uniform, potentially resulting in ‘clumped’ egg distributions in the soil and uneven egg ratios around neighboring plants. However, laboratory observations using the same batches of eggs showed that both species hatched at nearly the same time. Therefore, egg hatch in the greenhouse was also expected to occur simultaneously for the two species. In the context of climate warming and areas of continuous corn production, we infer that WCR populations may have the chance to expand northward into areas where their eggs previously could not overwinter, but where NCR eggs historically survived due to greater cold tolerance [14]. Such northward expansion of WCR could potentially challenge the local NCR populations. For example, NCR was the primary pest in Minnesota until the WCR arrived in the early 1960s [45]; yet, WCR now dominates in some parts of southern Minnesota [46]. Such shifts may alter species composition or even lead to displacement of NCR in some regions where it has historically been dominant [35]. Because WCR has evolved widespread resistance to multiple Bt proteins and insecticides, whereas documented resistance in NCR remains limited, a transition toward WCR-dominated populations in the future may reduce the overall effectiveness of current Bt corn hybrids, increase control costs, and necessitate more complex integrated pest management strategies [38,39,40].

Additionally, our results suggest that NCR are more negatively affected by interspecies larval competition, as the observed ratio of adult NCR to WCR in both diapause and non-diapause groups was significantly less than the expected ratio. Although these patterns were observed under controlled greenhouse conditions, they may have important implications for scouting, setting economic thresholds, and making management decisions in fields where both species coexist, particularly given the widespread Bt resistance in WCR and the emerging resistance in NCR [37,38,39,40,41]. For example, reduced selection pressure could potentially be placed on NCR due to larval root-tip feeding [44] and additional mortality caused by competition with WCR in the fields, which could help slow down the resistance development to Bt proteins in NCR.

In the first trial, we found that non-diapause populations of both WCR and NCR exhibit higher survival and adult emergence rates than their diapause counterparts, suggesting that CRW populations may trade off fitness for increased resilience to adverse winter conditions. Nonetheless, this pattern was not seen in the second trial. Although non-diapausing populations do not occur in the field for both CRW species, they were included in this study as a comparison to assess larval competitive potential without the confounding effects of winter mortality or variability in diapause termination. In the field, adult CRW lay eggs into the soil [22,47,48], and these eggs undergo diapause for 5 to 6 months to overcome harsh winter conditions before hatching in early summer the following year [35,49,50]. Under warmer temperatures and milder winters, overwintering mortality of CRW eggs may be reduced, potentially leading to increased CRW densities in the subsequent year [51]. Previous studies have demonstrated that increased CRW density intensifies larval competition, resulting in higher mortality, smaller adult size, shorter lifespans, reduced fecundity, delayed development, and diminished flight capacity [29,32,52,53].

Intraspecific competition has been documented in NCR populations [27,31], and Woodson reported intraspecific competition in WCR as larval density increased around the root zone [9]. Although our study did not exclusively focus on assessing intraspecific competition, we observed, to some extent, the indications of such competition within CRW populations. For example, the survival rates of WCR in the 100% D-WCR treatment were either numerically or statistically lower than those WCR in the 50% and 75% D-WCR treatments. In addition, competitive interactions can shape population structure, including adult emergence patterns and sex ratios. In field conditions, CRW males typically emerge earlier than females with peak male emergence occurring before peak female emergence [18,19], and females begin mating within hours after emergence [17]. Changes in sex ratio can significantly impact mating potential and overall species dynamics [21,35]. However, the sex composition of CRW could be affected by numerous other factors, including the type of corn planting (rotated versus continuous), monitoring methods and timing, and the type and placement of traps used [18,54,55,56,57,58]. For example, Weiss et al. observed a balanced 50:50 sex ratio in fields with low larval density and minimal root injury [32]. In contrast, using yellow sticky traps or hand collections, Godfrey and Turpin reported a higher proportion of females in first-year corn fields compared to continuous corn systems. They attributed this phenomenon to sex-biased interfield dispersal, as the only way the pre-rotation-resistant WCR could reach rotated corn was through flight, and females are much more likely to engage in interfield flight than males [59].

In this study, we used both diapausing and non-diapausing laboratory strains that have been maintained in the lab for many years. Long-term rearing may lead to laboratory selection and genetic bottlenecks, resulting in insects that may differ genetically and phenotypically from field populations. Consequently, findings based on laboratory strains may not fully reflect the responses of field-collected insects. It is also possible that prolonged laboratory confinement reduced the competitive ability of the NCR population used in this study. It has been documented that there is high variability among NCR populations, and among individual females within populations, in the proportion of eggs capable of remaining viable for more than one winter [60]. As a result, larval densities in a given year may need to account for contributions from both current oviposition and previously laid eggs in the soil. Although extended diapause was negligible in the selected NCR population used in this study, this trait is likely to play an important role in mediating interspecific interactions and competitive outcomes of CRW under field conditions. Therefore, future research should prioritize testing field-collected WCR and NCR populations from multiple geographic regions.

In summary, competition between NCR and WCR offers valuable insights into integrated pest management. Although both species share many biological similarities, field-evolved resistance to transgenic Bt-RW traits has been reported more frequently in WCR than in NCR [37,40,41,61,62,63,64,65,66]. Additionally, both species have evolved crop rotation resistance, with WCR laying eggs in non-corn hosts and NCR relying on extended diapause [67,68]. Although NCR populations have competitive survival and fitness in severely cold environments due to their greater tolerance of cold temperatures compared to WCR [14], climate warming may enable WCR to disperse and expand its range into these more northern latitude production areas thereby intensifying pest management challenges in corn production [69]. However, under certain environmental conditions unfavorable to both species, such as excessive rainfall leading to increased larval or pupal mortality or epizootic events, a portion of the NCR population remains as unhatched eggs in the soil to allow population recovery once conditions improve, whereas WCR populations are likely to be eliminated during vulnerable larval stages. In such cases, NCR’s extended diapause strategy may provide a survival advantage, allowing it to persist in the absence of WCR competition and potentially expand its range. Integrating insights from corn rootworm competition, biology, resistance development, and climate change is essential for making informed future management decisions to effectively mitigate CRW injury in agroecosystems [18,35,70].

## 4. Material and Methods

### 4.1. Source of Insects

Eggs of diapause and non-diapause WCR and non-diapause NCR insect populations were sourced from colonies maintained at the USDA-ARS laboratory in Brookings, SD. Diapause NCR eggs were obtained from Crop Characteristics, Inc. (Farmington, MN, USA). The diapause NCR colony has been maintained under laboratory conditions for multiple generations and propagated to retain only single-year diapause individuals, such that the frequency of extended diapause eggs is negligible. In addition, the non-diapause population of both WCR and NCR were developed from their respective diapause populations through selecting and propagating individuals that failed to enter diapause, ensuring that the diapause and non-diapause populations within each species share the same genetic background [70,71]. Furthermore, both WCR and NCR populations were collected prior to the commercialization of Bt rootworm (Bt-RW) proteins in 2003 and have been documented as susceptible to Bt proteins in current commercial corn hybrids (W. French, Personal communication).

All eggs were stored at 8 °C to maintain dormancy. One week before greenhouse infestation, they were transferred to 25 °C. Mature eggs were placed on moistened filter paper and closely monitored for embryonic development. Eggs were deemed suitable for experiments when body segmentation and mouth parts of the embryo became clearly visible under a microscope. The specified number of eggs of diapause WCR (thereafter refers to D-WCR), non-diapause WCR (thereafter refers to ND-WCR), diapause NCR (thereafter refers to D-NCR), and non-diapause NCR (thereafter refers to ND-NCR) for each treatment as detailed in Table 4, were prepared to study the interactions between WCR and NCR. The eggs were prepared in batches of hundreds based on the infestation egg ratio, then placed on moist soil in 30 mL plastic cups (Dart Container Corporation, Mason, MI 48854, USA). Corn plants grown in the greenhouse were infested with CRW eggs at the V2–V3 growth stage. In total, five different infestation ratios, as specified in Table 4, were used to examine the inter-specific dynamics between these insect groups.

### 4.2. Corn Plants

Seeds of non-Bt field corn used in this study were selected from the refuge seeds in a bag of hybrid DKC47-54RIB (VT Double PRO) obtained from Bayer CropScience (St. Louis, MO, USA). To remove insecticidal seed treatments, the corn seeds underwent a thorough washing process with tepid soapy water in the laboratory, following the method described by Gassmann et al. [37]. After washing, the seeds were naturally air-dried before further use. The cleaned, dried non-Bt corn seeds were planted in 5-L translucent round deli containers (Webstaurant store, Lititz, PA 17543, USA) and filled with a mixture of 1:1 Pro-mix BRK (Premier Horticulture Inc. Quakertown, PA, USA) and Sungro professional growing mix (Sun Gro Horticulture Distr. Inc. Agawam, MA, USA). Each pot was planted with three corn seeds and placed on tables in a greenhouse located at the University of Minnesota-St. Paul campus. Five days after emergence, one corn seedling per pot was carefully selected for uniform growth, while the remaining seedlings were thinned. Corn plants were maintained at a 14:10 L:D cycle, 25 °C temperature, and 70% relative humidity. The corn plants were appropriately fertilized and watered as needed to ensure optimum growth. To confirm the absence of commercially available rootworm Bt proteins, leaf tissues from randomly selected plants in the greenhouse were tested using QuickStick (EnviroLogic, Portland, ME, USA) test kits.

### 4.3. Laboratory Egg Hatch Rate

Prior to greenhouse infestation, the egg hatch rate of the four CRW populations was evaluated in the laboratory using a randomized complete block design (RCBD) with three replications per population. For each replication, 100 eggs were randomly selected. The CRW eggs were closely monitored in the laboratory and hatched neonates were counted daily.

### 4.4. Greenhouse Infestation

The first trial was initiated on 15 January 2024, with CRW adult emergence occurring from 21 February to 22 March 2024. The second trial was infested on 13 March 2024, with CRW adult emergence recorded from 15 April to 13 May 2024. For each trial, pots were infested on the 15th day after planting (approximately V2–V3 growth stage) with predetermined numbers of NCR and WCR eggs as detailed in Table 4.

**Figure 1:**
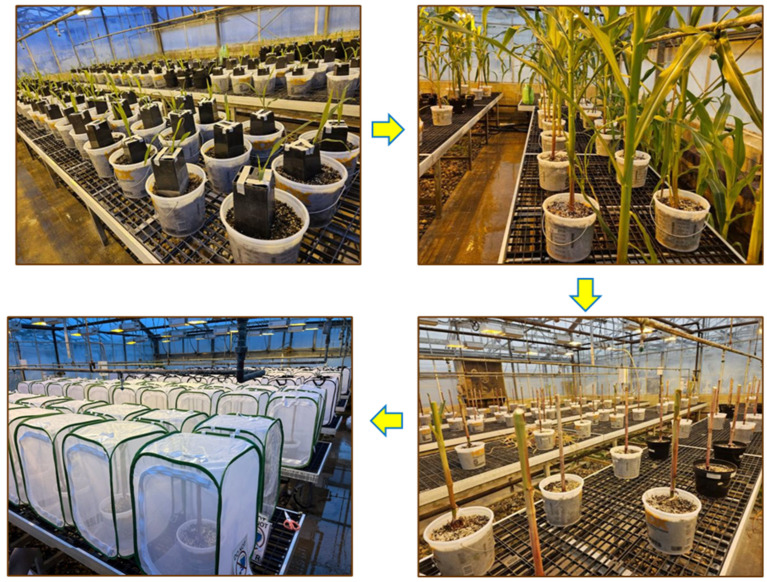
Corn planting, corn rootworm infestation, and plants trimmed to a height of 75 cm to accommodate the insect screen cages in the greenhouse.

Greenhouse infestations were initiated concurrently using the same egg batches as those used for the above laboratory egg hatch assays and conducted after microscopic examination confirmed active embryonic development and imminent hatching. In species-blended treatments, eggs from different populations were first thoroughly mixed in the laboratory before being randomly allocated to infestation pots. Each pot infestation involved digging four evenly spaced holes, each 5 cm deep and 5 cm from the center of the corn plant. Into each hole, 25 eggs were carefully placed and lightly covered with soil. To prevent egg desiccation, the soil surface was kept at a proper moisture after infestation. The processes of identifying, collecting, counting and infesting suitable eggs were completed within a single day. A temperature-dependent model for egg hatch, based on the greenhouse environmental conditions, was used to estimate the peak hatch and emergence period [72]. On the 20th day after egg infestation, corn plants were trimmed to approximately 75 cm in height to accommodate insect screen cages. Each pot was individually enclosed in a 92 × 61 × 61 cm insect screen cage in which one side consisted of a PVC/vinyl panel and the remaining sides were constructed of polyester mesh (Restcloud Insect and Butterfly Habitat Cage Terrarium, purchased from the Restcloud store on Amazon) to collect the emerging adult CRW (Figure 1). Adults were collected daily and recorded by species until no more adults were recovered. Overall, the experiment consisted of 10 treatments (detailed in Table 4) and was organized in a randomized complete block design. Each treatment was replicated with five blocks, with two pots in each block. As a result, a total of 100 pots were planted within the greenhouse for each trial. However, during the experiment, some pots were damaged by mice, resulting in some missing data and an effective range of three to five blocks for the treatments.

### 4.5. Data Analysis

The percentage of egg hatch was computed by dividing the total number of neonates by the total number of eggs, multiplied by 100. The percentage of adult emergence was calculated by dividing the total number of adults that emerged by the total number of infested eggs, multiplied by 100. The egg hatch rate and percentage of adult emergence were analyzed using a one-way ANOVA with rootworm population as the main factor, respectively, using SAS Institute 2023 [73]. To meet the normality assumption for the ANOVA test, the original data on the egg hatch rate and percentage of adult emergence were arcsine square-root transformed before analysis. Treatment means were separated using Tukey’s HSD test at α = 0.05 level. The observed survival of WCR and NCR in mixed-population treatments was corrected based on the survival of the corresponding 100% WCR and 100% NCR treatments using Abbott’s formula [74]. The observed ratio (N:W) was calculated by dividing the number of surviving NCR individuals by the number of surviving WCR individuals based on observed survival. The expected ratio (N:W) was calculated based on the initial egg infestation numbers for each species. For example, in treatment B, the ratio of NCR eggs to WCR eggs was 75:25, which is equivalent to 3:1. A *t*-test was then used to determine whether the species ratios of emerging adults differed from the expected species ratios based on the infestation levels of diapause and non-diapause NCR and WCR eggs. Degrees of freedom (DF) were determined by the number of replications used in the analysis.

## Figures and Tables

**Table 1:** The egg hatch rate (±SE) for four populations of diapause and non-diapause NCR and WCR eggs in laboratory for trials 1 and 2.

Insect Population ^a^	Mean Corn Rootworm Egg Hatch (%) ^b^	
	Trial 1	Trial 2
D-NCR	23.33 ± 1.20	28.33 ± 3.18
D-WCR	35.33 ± 6.67	29.00 ± 2.00
ND-NCR	32.67 ± 7.26	29.00 ± 2.65
ND-WCR	45.00 ± 3.61	38.00 ± 3.61
ANOVA (*F*, *p*)	*F*_3,8_ = 2.85; *p* = 0.1053	*F*_3,8_ = 2.50; *p* = 0.1330

^a^ D-NCR refers to diapause northern corn rootworm; D-WCR refers to diapause western corn rootworm; ND-NCR refers to non-diapause northern corn rootworm and ND-WCR refers to non-diapause western corn rootworm. ^b^ Egg hatch statistical comparisons are made within each trial (column) and not between trials (rows).

**Table 2:** The mean percentage of adult corn rootworm emergence (±SE) across 10 treatments with different ratios of NCR and WCR eggs in the greenhouse for trials 1 and 2. Mean values followed by the same letter within a trial (column) are not significantly different (*p* > 0.05; Tukey’s HSD test).

Trial	Population	Treatment	NCR:WCR ^a^	Mean Adult Emergence (%) ^b^	N ^c^	NCR (n) ^d^	WCR(n) ^d^
1	Diapause	A	D100:0	6.00 ± 0.75 a	4	48	0
		B	D75:25	6.90 ± 0.85 ab	5	36	33
		C	D50:50	10.70 ± 1.60 b	5	0	107
		D	D25: 75	18.25 ± 1.35 cd	4	3	143
		E	D0: 100	8.90 ± 1.28 ab	5	0	86
	Non-diapause	F	ND100:0	5.13 ± 0.69 a	4	41	0
		G	ND75:25	18.44 ± 1.29 cd	5	14	152
		H	ND50:50	16.00 ± 1.57 c	5	1	143
		I	ND25:75	21.90 ± 1.81 d	5	0	219
		J	ND0:100	20.00 ± 1.64 d	5	0	200
2	Diapause	A	D100:0	12.40 ± 2.08 abc	5	124	0
		B	D75:25	11.8 ± 2.90 abc	4	75	43
		C	D50:50	19.50 ± 1.77 d	5	75	120
		D	D25:75	18.10 ± 1.29 cd	5	21	160
		E	D0:100	10.80 ± 1.38 ab	5	0	108
	Non-diapause	F	ND100:0	8.67 ± 1.67 ab	3	26	0
		G	ND75:25	7.71 ± 1.80 ab	4	11	43
		H	ND50:50	6.75 ± 1.80 a	4	4	23
		I	ND25:75	9.50 ± 2.43 ab	5	3	54
		J	ND0:100	14.20± 2.50 cd	5	0	142

^a^ NCR: WCR indicates number of eggs infested for each species in each treatment. For example, in treatment A, D100:0 means infestation of 100 diapause NCR eggs and 0 diapause WCR eggs. ^b^ Adult emergence comparisons are made within each trial (column) and not between trials (rows). ^c^ N refers to the number of replications for each treatment. ^d^ n refers to the total number of adult corn rootworms (NCR or WCR) collected per treatment from cages in the greenhouse, categorized by diapause or non-diapause groups.

**Table 3:** The observed and expected NCR:WCR adult emergence ratios (±SE) for diapause and non-diapause populations in greenhouse studies.

Trial	Population	Treatment	NCR:WCR ^a^	Observed Ratio ^b^	Expected Ratio ^c^	DF	*p*-Value
1	Diapause	A	D100:0	-	-	-	-
		B	D75:25	1.8910 ± 0.9460	3	4	0.306
		C	D50:50	0.0000 ± 0.0000	1	4	<0.0001
		D	D25:75	0.0375 ± 0.0237	0.3333	3	0.0011
		E	D0:100	-	-	-	-
	Non- diapause	F	ND100:0	-	-	-	-
		G	ND75:25	0.4796 ± 0.1343	3	4	<0.0001
		H	ND50:50	0.0278 ± 0.0278	1	4	<0.0001
		I	ND25:75	0.0000 ± 0.0000	0.3333	4	<0.0001
		J	ND0:100	-	-	-	-
2	Diapause	A	D100:0	-	-	-	-
		B	D75:25	1.9560 ± 0.8010	3	3	0.2832
		C	D50:50	0.5730 ± 0.1360	1	4	0.0349
		D	D25:75	0.1160 ± 0.0170	0.3333	4	0.0002
		E	D0:100	-	-	-	-
	Non-diapause	F	ND100 :0	-	-	-	-
		G	ND75:25	0.3131 ± 0.1123	3	3	0.0002
		H	ND50:50	0.2936 ± 0.1817	1	3	0.0302
		I	ND25:75	0.0560 ± 0.0560	0.3333	4	0.0078
		J	ND0:100	-	-	-	-

^a^ NCR: WCR indicates number of eggs infested for each species in each treatment. For example, in treatment A, D100:0 means infestation of 100 diapause NCR eggs and 0 diapause WCR eggs. ^b^ Observed ratio (N:W) was calculated by dividing the number of surviving NCR individuals by the number of surviving WCR individuals based on observed survival. ^c^ Expected ratio (N:W) was calculated based on the initial egg infestation ratios. For example, in treatment B, the ratio of NCR eggs to WCR eggs was 75:25, which is equivalent to 3.

**Table 4:** Treatment groups consisting of varying numbers of NCR and WCR corn rootworm eggs from diapause and non-diapause populations used in the competition studies *.

Treatment	No. Eggs of D-NCR	No. Eggs of D-WCR	No. Eggs of ND-NCR	No. Eggs of ND-WCR
A	100	0	-	-
B	75	25	-	-
C	50	50	-	-
D	25	75	-	-
E	0	100	-	-
F	-	-	100	0
G	-	-	75	25
H	-	-	50	50
I	-	-	25	75
J	-	-	0	100

* D-NCR refers to diapause northern corn rootworm; D-WCR refers to diapause western corn rootworm; ND-NCR refers to non-diapause northern corn rootworm; and ND-WCR refers to non-diapause western corn rootworm.

## Data Availability

Data are contained within the article.
